# State Health Department Communication about Long COVID in the United States on Facebook: Risks, Prevention, and Support

**DOI:** 10.3390/ijerph19105973

**Published:** 2022-05-14

**Authors:** Linnea I. Laestadius, Jeanine P. D. Guidry, Andrea Bishop, Celeste Campos-Castillo

**Affiliations:** 1 Zilber School of Public Health, University of Wisconsin-Milwaukee, Milwaukee, WI 53205, USA; 2 Robertson School of Media and Culture, Virginia Commonwealth University, Richmond, VA 23284, USA; guidryjd@vcu.edu; 3 Department of Political Science, University of Wisconsin-Milwaukee, Milwaukee, WI 53211, USA; afbishop@uwm.edu; 4 Department of Sociology, University of Wisconsin-Milwaukee, Milwaukee, WI 53211, USA; camposca@uwm.edu

**Keywords:** COVID-19, long COVID, social media, health departments, social networks, Facebook, health communication

## Abstract

Greater public awareness of long COVID severity and susceptibility is needed to support those with long COVID and encourage preventive behaviors. It is not yet known to what extent health departments have informed the public about long COVID risks or offered guidance and support for those with long COVID. The objective of this research was to determine how and to what extent US state health departments have communicated with the public about long COVID via Facebook. Facebook posts with COVID-19 and long COVID terms made by 50 US state health departments plus Washington, DC, from 1 January 2020 to 31 January 2022, were collected using CrowdTangle. The first long COVID post appeared on 15 July 2020. From 15 July 2020 to 31 January 2022, state health departments made 49,310 COVID-19 posts and 137 long COVID posts. Using quantitative content analysis methods, long COVID posts were coded for health belief model constructs. Among long COVID posts, 75.18% included language about susceptibility, 64.96% severity, and 64.23% benefits of prevention. Cues to preventive action appeared in 54.01% of posts. 19.71% of posts provided guidance for those with long COVID. While health departments posted extensively about COVID-19, posts about long COVID were rare. This represents a missed opportunity to bolster arguments for preventive behaviors and support those experiencing long COVID.

## 1. Introduction

With almost 80 million cases of COVID-19 in the US and 480 million cases worldwide by the end of March 2022, the burden of post-COVID-19 conditions lasting four or more weeks has become a significant medical, social, and economic concern [1,2]. These conditions are most commonly known as long COVID, a term that was first used by Dr. Elisa Perego of Lombardy, Italy, in May of 2020, and that represents one of the first illnesses named and recognized through “patients finding one another on Twitter and through other social media” [3]. In December 2020, the US National Institute of Allergy and Infectious Diseases (NIAID) held the first federal workshop on “long COVID” [4], and by February 2021, Dr. Fauci, Director of the US NIAID, announced that the US government would begin using the research term “post-acute sequelae of SARS-CoV-2 infection (PASC)” [5]. The World Health Organization recognizes the seriousness of long COVID and suggests that the best way to prevent it is by “doing everything you can to avoid getting infected with the COVID-19 virus” [6]. Recent research by Antonelli et al. also suggests that two doses of the COVID-19 vaccine may decrease the odds of developing long-COVID symptoms [7]. 

Although the specific mechanisms of long COVID are still being examined, symptoms are now known to include fatigue, shortness of breath, cognitive symptoms, body pains, anosmia, abdominal issues, and anxiety/depression [8]. Females and those with pre-existing conditions may be at elevated risk of developing the condition [9,10]. As many as one in three adults may experience long COVID symptoms lasting months after initial SARS-CoV-2 infection [8,11], creating significant distress and disability. Adolescents and children also appear susceptible to long COVID [8,12]. Although multispecialty long COVID clinics have formed at many major medical centers, there is still no clear treatment for long COVID [1,13].

Despite this recognition by governments and researchers, patients experiencing long COVID have struggled with physician skepticism and confusion about their symptoms [9,14,15,16]. Limited understanding from family members, friends, and employers also complicates the emotional and practical aspects of recovery from long COVID, whereby long-haulers face stigma from experiencing a chronic yet “invisible” illness, as well as expectations that they return to work after a brief recovery period, despite current estimates that lingering symptoms can continue for months [17,18]. As with contested illnesses such as myalgic encephalomyelitis/chronic fatigue syndrome, somatic symptoms of long COVID have sometimes been dismissed and stigmatized as psychological [18,19,20]. Slowing progress toward overcoming these barriers is continued limited public concern and awareness about long COVID. For example, 49% of US adults remained unworried about long COVID in January 2022 [21]. 

In addition to complicating support for those with long COVID, failure to recognize the severity of long COVID may also limit the adoption of protective behaviors such as mask wearing, social distancing, and vaccination. The health belief model (HBM), which was developed to guide both health promotion and disease prevention efforts, posits that perceived severity, susceptibility, benefits, barriers, self-efficacy, and cues to action, are key in changing health-related behavior [22,23]. The HBM has been used in many health-focused quantitative content analyses [24,25,26], as well as health behavioral messaging studies [27,28,29]. As applied to COVID-19 and long-COVID prevention [30], the HBM indicates that effective communication should stress not just the severity of the condition, but also susceptibility to long COVID, the benefits of preventive measures, address known barriers to such preventive measures, and offer cues to action to encourage adoption of preventive behaviors. In short, greater public understanding of long COVID would benefit not just those who already have long COVID, through reduced stigma and greater awareness of symptoms and needs, but may also be a valuable strategy for encouraging preventive behaviors to limit COVID-19 spread and further long-COVID cases. 

Social media has become a critical means through which public health bodies communicate directly with the public [31], with the public in turn increasingly relying on social media for information about the COVID-19 pandemic. After the US Centers for Disease Control and Prevention (CDC), state health departments are the most frequently “followed” source of social media health information in the US [32]. Facebook, in particular, is an important conduit of health communication, as it remains the most widely used social media platform among US adults after YouTube [33]. The number of “followers” for US state health department Facebook pages also increased by 200%, following the federal COVID-19 emergency declaration in March 2020, with more total followers than on Twitter or Instagram [34]. Health departments are also more active on Facebook than on YouTube [35]. However, little is currently known about how, or even to what extent, US state health departments have messaged regarding long COVID on Facebook. 

This represents a significant gap in the literature given evidence of the severity of long COVID and current polling data indicating limited public concern about long COVID. Social media has also been recognized as a critical site for public health research, and a growing body of literature has focused on analyzing social media messaging by health departments and health agencies to determine appropriateness and provide context for understanding public behaviors [36,37]. At the time of writing, only one prior study had examined social media communication by health professionals of any kind about long COVID [38]. This study found that medical experts represented a small minority of posts about long COVID on YouTube, with the majority of posts made by news media and members of the public, suggesting a missed opportunity for professionals to connect with the public and share information. Health department communications remain unexamined. Therefore. this exploratory study examined Facebook posts from state health departments to determine (1) the number of posts that are about long COVID relative to the number of posts about COVID-19 more broadly, (2) how long COVID has been portrayed with regard to the health belief model, and (3) the types of informational resources offered to those who already have long-COVID symptoms. 

## 2. Materials and Methods

US state health department Facebook pages were identified using a list of agency names from the US Centers for Disease Control and Prevention list of state and territorial health department websites [39]. Using Facebook’s public insights tool, CrowdTangle [40], a list of Facebook pages of state health departments in all states plus Washington, DC, was created. The state of Massachusetts did not have a standalone Facebook page for its health department, so the main page for its government was used. This list was searched for posts with long COVID terms in text or images made from 1 January 2020 to 31 January 2022. Long COVID was defined broadly to capture any mentions of lasting or long-term health effects: "long-COVID" OR "post-acute COVID” OR "chronic COVID” OR "PASC" OR "post-acute sequelae of COVID-19" OR “post-COVID” OR “persistent COVID” OR “long-haul COVID” OR “lasting COVID” OR “long-term COVID” OR “lingering COVID” OR “long haul” OR “long-hauler” OR “long-haulers” OR “long-term symptoms” OR “lasting symptoms” OR "lingering symptoms" OR “persistent symptoms” OR “long-term effects” OR “lasting effects” OR “lingering effects” OR “persistent effects” OR “long-term side effects” OR “lasting side effects” OR “lingering side effects” OR “persistent side effects” OR “Voices of Long COVID” OR “post viral”. Use of a dash or not in search terms did not impact results. This search yielded 186 posts, which were downloaded as a CSV file of post data and links. To determine the overall volume of COVID-19 posting during that same time period, a second search was run from the same list of accounts using general COVID-19 terms (COVID OR coronavirus OR “SARS-CoV-2”), yielding 62,047 posts. The study did not require Institutional Review Board review, as it did not involve data on human subjects.

Long-COVID posts were manually screened for relevancy to long COVID and then coded in Microsoft Excel using content analysis procedures and a codebook informed by health belief model constructs [30]. More specifically, posts were coded for content related to long-COVID severity and susceptibility, as well as the benefits of preventive measures (masking, hand washing, social distancing, vaccination, etc.) and barriers to such measures, and cues to action to encourage the adoption of preventive behavior. Additionally, images, videos, and text were coded to identify the age group presented as being at risk, and posts and website links in posts were coded for the presence of information intended to support those with long COVID. Posts were also coded for the term used to refer to long COVID and any mentions of organized advocacy campaigns to support those with long COVID. Table 1 provides a summary of major codes and samples of coded content.

A random sample of 20% of posts (*n* = 37) was double-coded by L.L. and J.G., with a Krippendorff’s alpha average of 0.82, indicating strong reliability. L.L. coded the remaining posts. After coding, 49 posts from the long-COVID search were determined to not be related to long COVID (e.g., posts related to other chronic conditions, only pertaining to the Multisystem Inflammatory Syndrome in Children, or to the COVID-19 pandemic more broadly but not long COVID) and were, thus, excluded from the results.

## 3. Results

**Figure 1:**
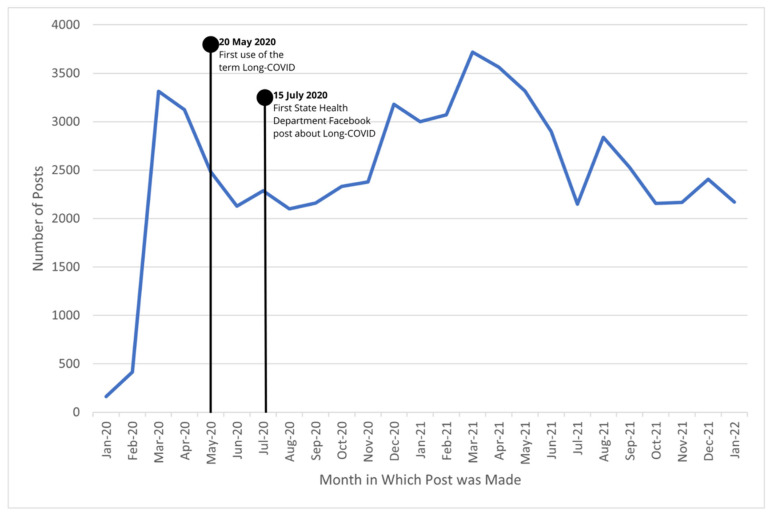
State health department COVID-19-related Facebook posts, 1 Jan 2020–31 Jan 2022.

**Figure 2:**
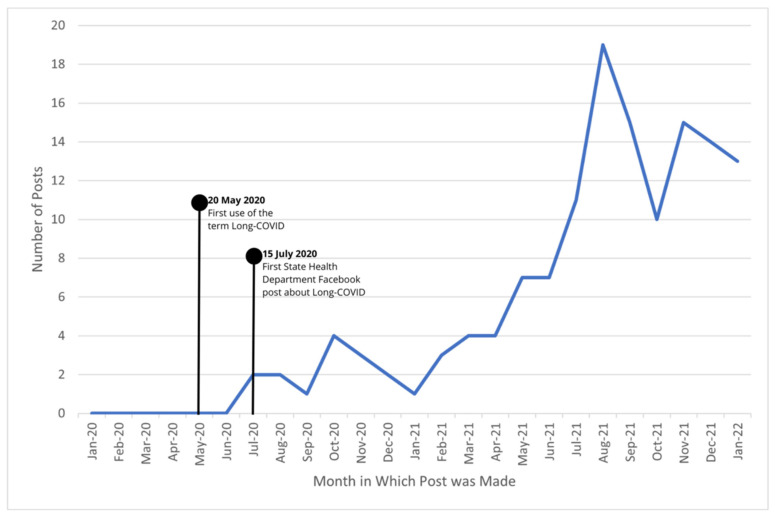
State health department long COVID-related Facebook posts, 1 Jan 2020–31 Jan 2022.

The first long-COVID-related post was made on 15 July 2020, by the Kansas Department of Health and Environment, stating COVID-19 can have long-term health effects. Between 15 July 2020 and 1 February 2022, health departments from all 50 states plus Washington, DC, made 49,310 posts related to COVID-19 (Figure 1). In the same time period, 137 posts from 32 health departments addressed long COVID in some capacity (Figure 2). Posts originated most frequently from North Dakota, Alaska, New York, and North Carolina, with 10 or more long COVID-related posts each. Supplemental Table S1 provides the number of posts from each state related to COVID-19 and long COVID. 

Among related posts, the term long COVID was used in 39.41% (*n* = 54) of posts. Long-haul COVID or long-haulers were mentioned in 24.82% (*n* = 34) of posts and PASC or post-acute sequelae of SARS-CoV-2 infection was mentioned in 1.46% (*n* = 2) of posts. Many posts did not make use of specific terms and simply addressed the long-term health effects of COVID-19 in more descriptive ways. For example, in a post from January 2022, the Hawaii State Department of Health explained that “The long-term effects of COVID include scarred lungs, brain fog, chest pains and fatigue. Don’t let COVID symptoms affect your peak performance for years to come. Mask up and get vaccinated.” Images, illustrations, or videos of people portrayed as at risk of COVID-19 infection were featured in 51.09% of posts (*n* = 70), with adults (45.26%, *n* = 62) more commonly depicted than children and adolescents (8.03%, *n* = 11). When considering texts as well as images, 18.24% of posts (*n* = 25) conveyed that children and adolescents are at risk for developing long COVID. Links to long-COVID-related information were provided in 42.34% of related posts (*n* = 58), with state agency websites most frequently linked (12.41%, *n* = 17), followed by the US CDC, news stories about long COVID, and medical and scientific organizations or journals (each at 9.49%, *n* = 13). 

Regarding framing of related posts, 75.18% (*n* = 103) included language suggesting that readers should be concerned about long-COVID susceptibility, and 16.06% (*n* = 22) included individuals sharing personal experiences with long COVID. For example, a December 2021 post from the Virginia Department of Health shared an image of and a quote from a person with long COVID, paired with an informational caption “‘I used to run 5 to 6 miles a day. Now, when I walk up a flight of stairs, I’m gasping for air... I’m telling my Long COVID story because don’t want it to ruin other people’s lives like it did mine.’—Rob, 22. Long-COVID survivors are sharing their stories so that others won’t have one to share. Many people, including young adults, have been infected with COVID-19 and continue to suffer from persistent health problems months later. Anyone can get Long COVID, but the best way to prevent it is to get vaccinated #VoicesofLongCOVID.”

Overall, 12 posts (8.76%) mentioned or linked to the “Voices of Long COVID” campaign. Three posts (2.19%) mentioned the group, SurvivorCorp. Almost 65% (*n* = 89) stressed the severity of long COVID through text or images, with 41.61% (*n* = 57) mentioning long-COVID symptoms. Benefits of preventing long COVID were mentioned in 64.23% of posts (*n* = 88), with vaccines mentioned most frequently (56.20%, *n* = 77), followed by mask wearing (9.49%, *n* = 13) and social distancing (5.84%, *n* = 8). Perceived barriers to long-COVID preventive measures were rarely addressed (8.76%, *n* = 12). Cues to preventive action (e.g., “get vaccinated”) were present in 54.01% of posts (*n* = 74). For individuals already experiencing long-COVID symptoms, 19.71% of posts (*n* = 27) mentioned or linked to some form of advice or guidance. This included information about support groups, new research on symptoms and treatments, guidance to speak with physicians, and, more rarely, guidance for employees and employers. As an illustrative example, the Wisconsin Department of Health Services posted in September 2021: “DYK? [Did you know?] Anyone who had #COVID19, including people who did not initially have symptoms, can get long COVID. There is support available. Find information and resources [link to state website].”

## 4. Discussion

The date of the first US state health department Facebook posts related to long COVID corresponds to when physicians began to publish about patterns of persistent symptoms following SARS-CoV-2 infection [41,42]. However, the volume of long-COVID posts since that time was extremely small, compared with the volume of posts made about COVID-19 more broadly, indicating that long COVID did not receive the attention it warrants. Further, several state health departments did not have a single Facebook post about long-COVID awareness or support despite posting about COVID-19 more generally. Although the lack of attention to long COVID by health departments cannot be causally linked to the limited public concern about long COVID, based on the data examined here, they are suggestive of an information environment in which public health leaders have chosen not to emphasize that COVID-19 infection may cause lasting health impacts and that hospitalization and death are not the only issues of concern [16].

Stories about individuals with long COVID were also relatively rare despite evidence from the tobacco control literature that personal narratives such as those shared in the US CDC’s *Tips From Former Smokers* campaign have been effective at promoting behavior change [43]. State health departments should draw upon communication lessons from other health-behavior-related issues. Future research should consider the impacts of personal long COVID stories on both preventive behaviors and support for those who already have long COVID. The small number of posts with informational resources for those currently suffering from long COVID also poses a concern given the challenges that those with persistent symptoms have faced in obtaining appropriate care and work accommodations [1,16,44]. The limited number of posts about long COVID may also help perpetuate the impression that otherwise healthy individuals will fully recover from COVID-19, making little effort to increase public understanding of how many people experience long COVID or the burden that it poses for long-haulers. An additional concern is the lack of posts that address perceived barriers to long COVID preventive measures, one of the relevant HBM measures in this case. A common perceived barrier to COVID-19 vaccination is belief in misinformation about COVID-19 vaccines [28] and social media platforms, including Facebook, have been fertile ground for such misinformation despite fact checkers’ corrective efforts [45]. State health departments should actively address and debunk misinformation about COVID-19 and long COVID.

Findings are also consistent with the limited prior research on health professional communication about long COVID on social media [38]. Taken together, it appears that many health professionals and agencies have ceded the social media space to news outlets, patient advocacy groups, and individuals sharing their own experiences with long COVID [46,47]. While these groups, particularly long-haulers themselves, have been able to use social media effectively to mobilize recognition of long COVID by medical communities and governments [3], the absence of official messaging may contribute to ongoing long COVID stigma and marginalization. It is currently unclear why long COVID has remained relatively neglected by state health department communication in the US. When US state health departments posted about long COVID and lingering COVID-19 symptoms, posts stressed susceptibility to and severity of long COVID, indicating some recognition of both the lingering risks of COVID-19 infection and the fact that long COVID may be a compelling message for encouraging preventive behaviors. Vaccination, in particular, was mentioned, although mentions of mask wearing and social distancing were notably rare despite an endorsement from both the American Medical Association and the World Health Organization as an effective strategy for preventing long COVID [6,48]. It is possible that limited attention to long COVID and to non-vaccine approaches reflects the influence of the US CDC and the White House COVID-19 Response Team, which have placed a primary emphasis on preventing severe disease and hospitalization rather than controlling the spread of COVID-19 more broadly [49]. Further research is needed to understand the drivers and barriers underpinning state-level COVID-19 communication pertaining to long COVID. 

## 5. Limitations

Data were limited to a single social media platform. Additionally, only posts made in English and from US agencies were analyzed. Given the significant inequities in COVID-19 cases by race and ethnicity in the US [50], and the fact that Spanish is the most commonly spoken language in the US after English [51], future studies should consider the Spanish language long COVID communication from health departments. Additionally, a comparative cross-national approach would yield valuable insights into the exploration of long COVID communication, particularly if paired with data on national perceptions of long COVID severity. Finally, there is some possibility that a state health department may have posted about long COVID without applying any of the terms used in these searches, although a comprehensive set of search terms was used to minimize this risk. 

## 6. Conclusions

While state health departments posted extensively about COVID-19 on Facebook, posts relating to long COVID were rare between 2020 and the start of 2022. Overall, this low volume of messaging suggests a missed opportunity for US state health departments to inform the public about risks and communicate positive messages that may promote uptake of preventive behaviors, including COVID-19 vaccines, given recent evidence suggesting they may reduce the odds of persistent COVID-19 symptoms [7]. Although HBM constructs were generally present, suggesting that posts may encourage the adoption of desired behaviors, the volume of posts, particularly when compared with the volume of posts about COVID-19 more generally, indicates that health departments did not find it a priority to communicate about long COVID. Therefore, the primary issue to be rectified regarding prevention is not so much the content of posts but rather the limited attention that long COVID has received overall. 

With regard to information serving those who already have long COVID, the issue is further compounded by appearing to be a marginal focus within what was already a marginalized communication issue. The paucity of messaging also meant limited information targeting those experiencing long COVID symptoms who may be unsure of where to seek assistance. Greater awareness of the severity and symptoms of long COVID may help reduce stigma from family members, friends, and employers and promote greater public and policymaker understanding of the differences between COVID-19 recovery and survival [16,18].

With several state health departments not messaging about long COVID in any capacity on Facebook, the social media platform where health departments have the most followers, there is a critical need for increased action. First, health departments that have yet to message on long COVID may wish to draw upon examples of messaging used by the health departments identified here. Additional steps may be needed to identify best practices, such as examining which messages elicited greater engagement from users. Second, third-party resources such as the “Voices of Long COVID” campaign may be able to offer health departments low- or no-cost messaging that has already been developed and tested. This may be particularly important as surveys suggest that one-third of US residents now find information from their state health department to be unreliable [52]. Thus, the use of trusted stakeholders and community voices is critical for COVID-19 communication of all types on social media [31]. Third, long-COVID patient advocacy groups may wish to focus attention on health departments as potential partners that could be persuaded to help disseminate messages. With as many as 31 million people in the US having developed long COVID as of January 2022, leading to over 1 million people being absent from the labor market at any given moment [53], it is increasingly clear that long-COVD communication should be considered a priority for health departments. As one of the ten essential public health services within the US is to “communicate effectively to inform and educate people about health, factors that influence it, and how to improve it,” [54] clearer long COVID communication is also well aligned with health department goals. 

## Figures and Tables

**Table 1:** Primary codes, definitions, and examples.

Code/Subcode	Definition	Example
Susceptibility to Long COVID	Posts suggesting that readers are at risk of developing long COVID	“Some studies and surveys with patients hospitalized with COVID-19 show that up to 50%-80% of people continue to have symptoms up to eight weeks after they contracted COVID-19, even though the virus is no longer in their bodies.” *Idaho Department of Health and Welfare*
Severity of Long COVID	Posts suggesting that long COVID would pose a serious risk to health and well-being	“For some people, the symptoms of long COVID can be quite debilitating and very, very similar to chronic fatigue syndrome symptoms…" *Alaska Health and Social Services*
Long-COVID Symptoms	Posts detailing the symptoms that people with long COVID may experience	“Most people who get sick with #COVID19 recover completely within a few weeks, but some continue to have lasting side effects like shortness of breath and headaches.” *Georgia Department of Public Health*
Long-COVID Experiences	Posts sharing a personal experience from someone who developed long COVID	“Before COVID I used to read all the time. Now I can’t even read a simple paragraph without getting tired and frustrated. With the help of my teachers and staff, they have been very supportive at my school. After all this time suffering, I strongly urge others to get the vaccine because it can save your life and it can make sure that you and others don’t have to get affected the same way and have to suffer like I have.” *Oregon Health Authority*
Benefits of Means of Preventing Long COVID	Posts describing methods to effectively reduce the threat of long COVID	“The best way to avoid getting long COVID is to protect yourself from COVID-19. Get vaccinated, wear a mask, physically distance, and wash your hands frequently.” *Wisconsin Department of Health Services*
Prevention Benefits/Masks	Posts describing masks as an effective strategy for reducing the threat of long COVID	“The long-term effects of COVID include scarred lungs, brain fog, chest pains and fatigue. Do not let COVID symptoms affect your peak performance for years to come. Mask up and get vaccinated.” *Hawaii State Department of Health*
Prevention Benefits/Social Distancing	Posts describing social distancing as an effective strategy for reducing the threat of long COVID	“COVID can have devastating long-term effects. So wear a mask. Wash your hands. Social distance. Don’t take the risk.” *New York State Health Department*
Prevention Benefits/Vaccines	Posts describing vaccines as an effective strategy for reducing the threat of long COVID	“Vaccinating drastically reduces your chance of contracting #longhaul #COVID19.” *Georgia Department of Public Health*
Barriers to Prevention	Posts addressing any barriers to engaging in long COVID preventive behaviors	“Because some people with #COVID19 can have very mild symptoms, some may see natural infection as preferable to receiving the COVID-19 vaccine. The fact is that natural immunity or protection from COVID-19 is not preferable to getting vaccinated. The risk of severe illness and death from COVID-19 outweighs the benefit of natural immunity… Also, scientists are still learning about the long-term effects of COVID-19, but some people continue to have some longer-term effects from their illness.” *Oregon Health Authority*
Cues to Action	Posts encouraging readers to take action to prevent long COVID	“For 1 in 4 COVID-19 patients, the fight doesn’t stop once the virus goes away. Protect yourself and your loved ones by getting vaccinated. #ctvaxfacts” *Connecticut Department of Public Health*
Informational Resources for People with Long COVID	Posts offering or linking to guidance, advice, or research targeting those who already have long COVID	“#COVID19 and recovering from it looks different for everyone. For some people, symptoms last for weeks, or even months. That’s called long COVID. Learn more about long COVID and what #DHSWI is doing to help those experiencing it” *Wisconsin Department of Health Services*

## Data Availability

Facebook posts are available via Facebook’s public insights tool CrowdTangle.

## Supplementary Materials

The following supporting information can be downloaded at: https://www.mdpi.com/article/10.3390/ijerph19105973/s1, Table S1: State counts of COVID-19 and long COVID US Health Department posts made between 15 July 2020 and 31 January 2022.
