# Supplementary material for: State Health Department Communication about Long COVID in the United States on Facebook: Risks, Prevention, and Support
Source: Int J Environ Res Public Health. 2022 May 14;19(10):5973. doi: 10.3390/ijerph19105973 (PMC9140570; doi:10.3390/ijerph19105973)
Supplement: Supplementary file 1 [file ijerph-19-05973-s001.zip › ijerph-1678606-supplementary.pdf]

Table S1. Number of COVID-19 and Long COVID Posts made by US State Health Departments between July 15, 2020 and January 31, 2022

**State Facebook Page**	**COVID-19 posts**	**Long COVID posts**
Alabama Public Health	1102	3
Alaska Health and Social Services	1822	12
Arizona Department of Health Services	792	0
Arkansas Department of Health	971	0
California Department of Public Health	959	0
Colorado Department of Public Health and Environment	2000	0
Connecticut Department of Public Health	307	1
DC Health	1216	5
Delaware Division of Public Health - DPH	1649	2
Florida Department of Health (DOH)	253	0
Georgia Department of Public Health	694	5
Hawaii State Department of Health	874	4
Idaho Department of Health and Welfare	1528	8
Illinois Department of Public Health (IDPH)	1050	3
Indiana Department of Health	728	7
Iowa Department of Public Health	232	0
Kansas Department of Health and Environment	350	3
Kentucky Department for Public Health	146	0
Louisiana Department of Health	1013	2
Maine CDC	287	1
Maryland Department of Health	593	0
Mass.gov	253	0
Michigan Department of Health and Human Services	784	1
Minnesota Department of Health	1373	2
Mississippi State Department of Health	808	0
Missouri Department of Health and Senior Services	400	1
Montana Department of Public Health and Human Services	12	0
NC Department of Health and Human Services	1400	10
Nebraska Department of Health and Human Services	346	0
Nevada Department of Health and Human Services	133	1
New Jersey Department of Health	1592	1
New Mexico Department of Health	1379	4

NH Department of Health and Human Services	253	1
North Dakota Department of Health	1049	14
NYSDOH - New York State Health Department	1247	11
Ohio Department of Health	503	0
Oklahoma State Department of Health	537	0
Oregon Health Authority	1141	6
Pennsylvania Department of Health	1266	1
Rhode Island Department of Health	2355	4
SC DHEC	2574	5
South Dakota Department of Health	761	0
Tennessee Department of Health	961	1
Texas Department of State Health Services	416	0
Utah Department of Health	136	0
Vermont Department of Health	764	1
Virginia Department of Health	645	6
Washington State Department of Health	2217	2
Wisconsin Department of Health Services	718	9
WV Department of Health & Human Resources	4603	0
Wyoming Department of Health - PHEP	118	0
